# Recombinant and endogenous ways to produce methylated phospholipids in *Escherichia coli*

**DOI:** 10.1007/s00253-021-11654-8

**Published:** 2021-10-28

**Authors:** Julia Kleetz, Georgios Vasilopoulos, Simon Czolkoss, Meriyem Aktas, Franz Narberhaus

**Affiliations:** Microbial Biology, Faculty of Biology and Biotechnology, Ruhr University Bochum, Bochum, Germany

**Keywords:** Phospholipids, Phosphatidylcholine, *Escherichia coli*, Membrane remodeling

## Abstract

*Escherichia coli* is the daily workhorse in molecular biology research labs and an important platform microorganism in white biotechnology. Its cytoplasmic membrane is primarily composed of the phospholipids phosphatidylethanolamine (PE), phosphatidylglycerol (PG), and cardiolipin (CL). As in most other bacteria, the typical eukaryotic phosphatidylcholine (PC) is not a regular component of the *E. coli* membrane. PC is known to act as a substrate in various metabolic or catabolic reactions, to affect protein folding and membrane insertion, and to activate proteins that originate from eukaryotic environments. Options to manipulate the *E. coli* membrane to include non-native lipids such as PC might make it an even more powerful and versatile tool for biotechnology and protein biochemistry. This article outlines different strategies how *E. coli* can be engineered to produce PC and other methylated PE derivatives. Several of these approaches rely on the ectopic expression of genes from natural PC-producing organisms. These include PC synthases, lysolipid acyltransferases, and several phospholipid *N*-methyltransferases with diverse substrate and product preferences. In addition, we show that *E. coli* has the capacity to produce PC by its own enzyme repertoire provided that appropriate precursors are supplied. Screening of the *E. coli* Keio knockout collection revealed the lysophospholipid transporter LplT to be responsible for the uptake of lyso-PC, which is then further acylated to PC by the acyltransferase-acyl carrier protein synthetase Aas. Overall, our study shows that the membrane composition of the most routinely used model bacterium can readily be tailored on demand.

**Key points**

*• Escherichia coli can be engineered to produce non-native methylated PE derivatives.*

*• These lipids can be produced by foreign and endogenous proteins.*

*• Modification of E. coli membrane offers potential for biotechnology and research.*

**Graphical abstract:**

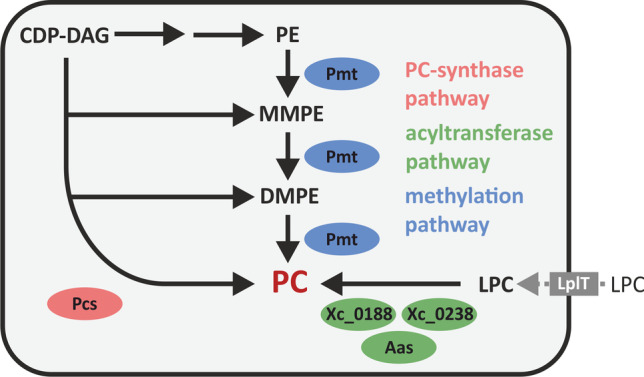

**Supplementary Information:**

The online version contains supplementary material available at 10.1007/s00253-021-11654-8.

## Introduction

All living cells are surrounded by membranes, which not only protect them from external threats but also are important for the controlled import and export of molecules. Principles of bacterial lipid membrane synthesis of Gram-negative bacteria mostly derive from studying the model organism *Escherichia coli*. The cytoplasmic membrane of *E. coli* consists mainly of three phospholipids: phosphatidylethanolamine (PE, 75%), phosphatidylglycerol (PG, 20%), and cardiolipin (CL, 5%) (Raetz and Dowhan 1990). Since *E. coli* is commonly found in the lower intestine of warm-blooded organisms, it is challenged by a wide range of constantly changing stress conditions, including nutrient limitation or exposure to varying pH, osmolarity, and temperature. Previous studies showed that cells actively re-organize their membrane lipids and fatty acids in order to counteract such stress factors. Fatty acid biosynthesis genes are regulated by nutrient availability and growth rate, resulting in changes of cell length and width (Tao et al. 1999). Under higher temperatures or acidic conditions, a considerable percentage of the unsaturated fatty acids is either replaced by saturated fatty acids or converted to cyclopropane derivatives (Brown et al. 1997; Sohlenkamp 2019). However, modifications of the membrane as part of the stress response are not limited to fatty acid moieties but also to the phospholipid composition. Increased osmotic pressure causes an accumulation of the phospholipid CL (Lusk and Kennedy 1972). Conversely, deficiency of PE or CL leads to increased heterogeneity of cell size as well as defected growth in minimal medium compared to the wild type (Rowlett et al. 2017). Recently, it was shown that the phospholipid composition within the cytoplasmic membrane of Gram-negative bacteria is highly asymmetric, dynamic, and cell cycle dependent (Bogdanov et al. 2020). Those results, among others, emphasize the plasticity of the *E. coli* membrane and its ability to adapt to changing conditions, which include challenges imposed by recombinant protein production and various biocatalytic processes (Gubellini et al. 2011; Opekarová and Tanner 2003).

**Fig. 1:**
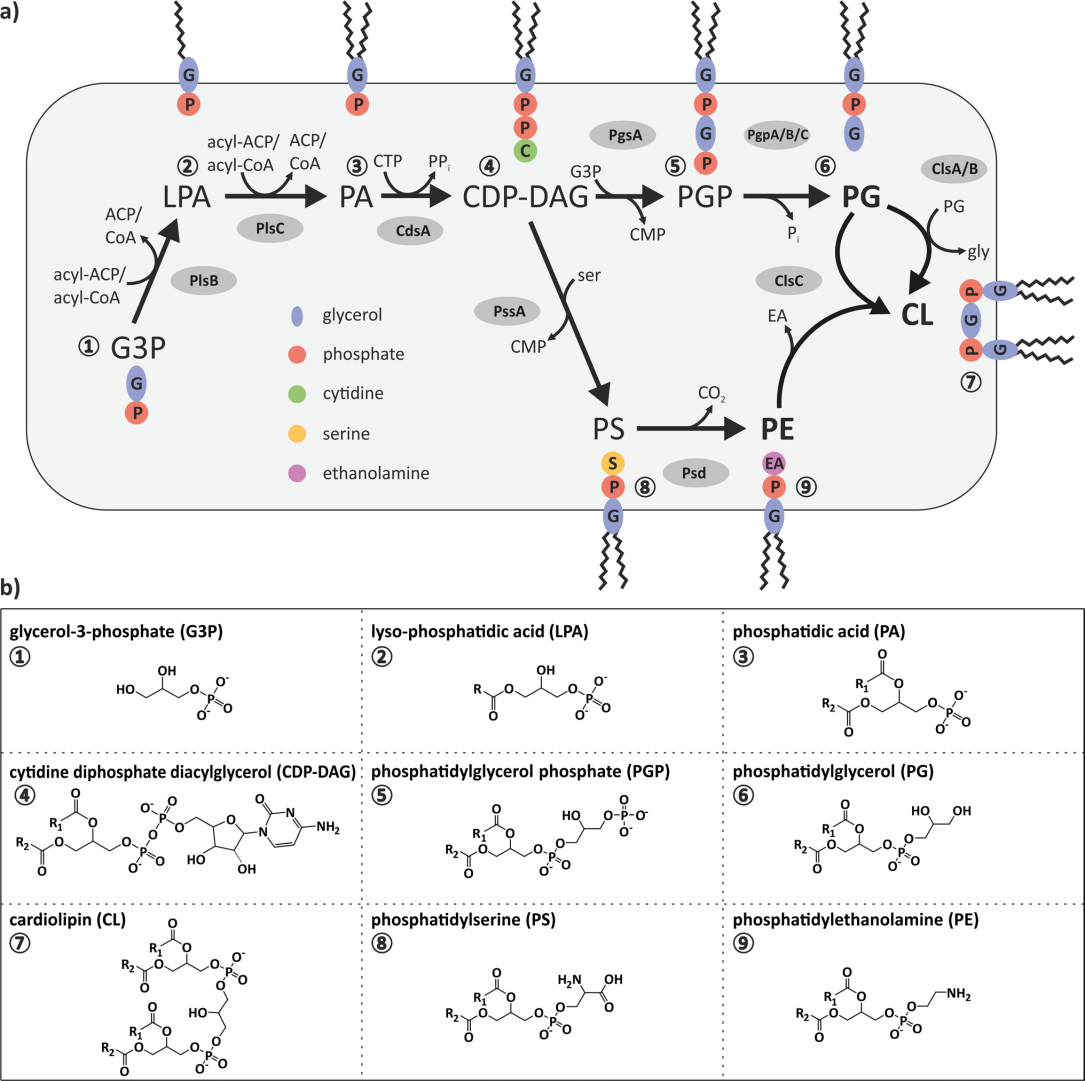
Overview of phospholipid-synthesis pathways in *E. coli* (**a**) and chemical structures of products and intermediates of the described pathways (**b**). Details on the individual enzyme reactions are given in the text. ACP: acyl carrier protein; CMP: cytidine monophosphate; CTP: cytidine triphosphate; ser: *L*-serine; gly: glycerol; R: alkyl-group

Phospholipid biosynthesis in *E. coli* starts with the acylation of glycerol-3-phosphate (G3P) via two subsequent steps to form lysophosphatidic acid (LPA) and phosphatidic acid (PA) (Fig. 1). These reactions are catalyzed by the activity of two acyltransferases, PlsB and PlsC, using acyl-ACP or acyl-CoA as acyl donor. Then, the CDP-DAG synthase CdsA converts PA and cytidine triphosphate (CTP) to cytidine diphosphate diacylglycerol (CDP-DAG), the central precursor of all phospholipids. For the synthesis of the most abundant zwitterionic phospholipid PE, CDP-DAG is condensed with *L*-serine to form the intermediate phosphatidylserine (PS) by the phosphatidylserine synthase PssA. The decarboxylase Psd catalyzes the decarboxylation of PS to PE. For synthesis of the anionic PG, CDP-DAG is condensed with G3P resulting in the intermediate PG phosphate (PGP) by the phosphatidylglycerol phosphate synthase PgsA. In a next step, PGP is dephosphorylated by the action of one of the three known PGP-dephosphorylases PgpA, PgpB, and PgpC, yielding PG (Lu et al. 2011). For CL synthesis, three different synthases (Cls) have been described in *E. coli.* ClsA or ClsB convert two PG molecules to CL, whereas ClsC utilizes a PG and a PE molecule to produce CL (Tan et al. 2012).

Bacterial membranes are generally believed to feature a simplistic lipid composition like *E. coli* with PE, PG, and CL. Only recently, it was recognized that the membrane composition of many bacteria is much more diverse than originally anticipated (Sohlenkamp and Geiger 2016). The human pathogen *Campylobacter jejuni* for example accumulates unusually large amounts of the lysophospholipids lyso-PE (LPE) and lyso-PG (LPG) (Cao et al. 2020). Other bacteria, like *Flavobacterium ummariense* or *Bdellovibrio bacteriovorus*, accumulate significant amounts of PS, which is usually directly converted to PE (Lata et al. 2012; Nguyen et al. 2008). In the notorious pathogen *Mycobacterium tuberculosis*, phosphatidylinositol (PI) plays an essential role for cell viability, even though PI is a typical eukaryotic phospholipid (Jackson et al. 2000) and only rarely a component of bacterial membranes.

The most abundant eukaryotic phospholipid phosphatidylcholine (PC) can be synthesized by an estimated number of ~ 15% of all bacteria (Geiger et al. 2013), with relative amounts ranging from 6% in *Xanthomonas campestris* to over 70% in *Acetobacter aceti* (Hanada et al. 2001; Moser et al. 2014). Recent studies have revealed numerous functional roles of PC for bacteria. A PC-deficient mutant of *Sinorhizobium meliloti* is unable to establish a productive nitrogen-fixing symbiosis with its host plant alfalfa (Sohlenkamp et al. 2003; Geiger et al. 2021). Similarly, decreased PC amounts in membranes of *Bradyrhizobium diazoefficiens* USDA 110 (formerly *Bradyrhizobium japonicum* (Delamuta et al. 2013)) cause a reduction of nitrogen-fixation activity of infected soybean root nodules compared to the wild type (Minder et al. 2001). In contrast, the symbiosis of *Bradyrhizobium* sp. SEMIA 6144 is not affected by decreased PC levels (Medeot et al. 2010). PC-deficient mutants of the crown-gall tumor-inducing plant pathogen *Agrobacterium tumefaciens* display several phenotypic changes, such as impaired motility, defects in response to elevated temperatures or sodium dodecyl sulfate (SDS), and loss of virulence (Klüsener et al. 2009; Wessel et al. 2006). Consistent with the loss of virulence in *A. tumefaciens*, the absence of PC impairs the virulence of the human pathogens *Brucella abortus* and *Legionella pneumophila* (Comerci et al. 2006; Conover et al. 2008; Conde-Alvarez et al. 2006).

**Fig. 2:**
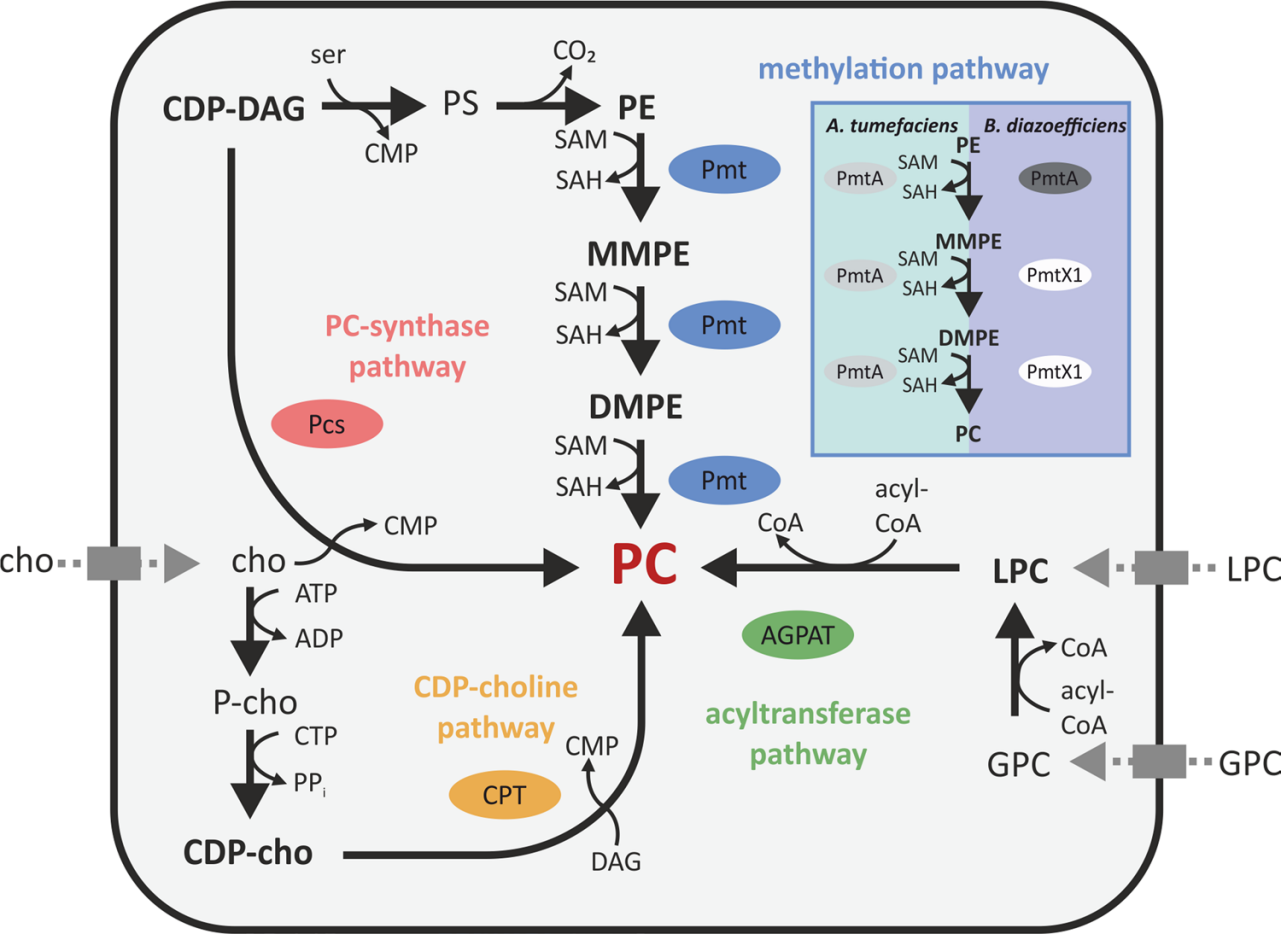
Different pathways for PC synthesis in bacteria. The PC synthase pathway (purple), methylation pathway (orange), CDP-choline pathway (blue), and acyltransferase pathway (green) are displayed with the corresponding substrates, products, and responsible enzymes. Specific examples of enzymes responsible for the methylation pathway in *A. tumefaciens* and *B. diazoefficiens* are displayed in the blue box. Details on the individual enzyme reactions are given in the text. CDP-DAG: cytidine diphosphate diacylglycerol; ser: *L*-serine; CMP: cytidine monophosphate; PE: phosphatidylethanolamine; MMPE: monomethyl-PE; DMPE: dimethyl-PE; LPC: lyso-PC; GPC: glycerophosphocholine; PC: phosphatidylcholine; cho: choline; P-cho: phosphocholine; Pmt: phospholipid *N*-methyltransferase; Pcs: PC synthase; CPT: CDP-choline:1,2-DAG choline phosphotransferase; CoA: coenzyme A; AGPAT: 1-acyl-*sn*-glycerol-3-phosphate acyltransferase

PC synthesis in bacteria typically occurs by either the methylation pathway or the PC synthase pathway. However, alternative CDP-choline-dependent or acyltransferase-dependent pathways have also been reported (Sohlenkamp and Geiger 2016) (Fig. 2). In the methylation pathway, phospholipid *N*-methyltransferases (Pmts) catalyze the *S*-adenosyl methionine (SAM)-dependent threefold *N*-methylation of the PE headgroup to yield monomethyl-PE (MMPE), dimethyl-PE (DMPE), and finally PC. Bacterial Pmts are cytosolic proteins and can be divided into two groups, the *Rhodobacter*-type and the *Sinorhizobium*-type based on amino acid sequence similarity (Sohlenkamp et al. 2003). The conversion of PE to PC is catalyzed by either a single Pmt as in *A. tumefaciens* (Aktas and Narberhaus 2009) and *S. meliloti* (de Rudder et al. 2000), or by the consecutive action of multiple enzymes, as in *B. diazoefficiens* (Hacker et al. 2008b). In *B. diazoefficiens*, the *Sinorhizobium*-type PmtA most efficiently methylates PE to MMPE and to a lesser extent MMPE to DMPE, while the *Rhodobacter*-type PmtX1 utilizes MMPE as a substrate to form DMPE and PC. Similar Pmts with diverse substrate and product preferences have recently been reported in several thermophilic bacteria (Kleetz et al. 2021).

The PC synthase pathway is exclusive to bacteria. Here, PC is synthesized via the condensation of CDP-DAG with choline by the PC synthase (Pcs). Pcs enzymes are integral membrane proteins that belong to the superfamily of CDP-alcohol phosphotransferases. This pathway depends on an exogenous choline source from the environment or from the host (de Rudder et al. 1999). Some bacteria, like *Pseudomonas aeruginosa* and *B. abortus*, have been described to synthesize PC solely via the Pcs pathway (Wilderman et al. 2002; Comerci et al. 2006), while others, such as *Zymomonas mobilis*, exclusively use the methylation pathway (Tahara et al. 1987). However, many bacteria utilize both pathways for PC synthesis, as described for *A. tumefaciens*, *B. diazoefficiens*, *Rhizobium meliloti*, or *Rhizobium leguminosarum* (Wessel et al. 2006; de Rudder et al. 1999; Martínez-Morales et al. 2003).

An uncommon pathway for PC synthesis in bacteria occurs in *Treponema denticola*, where PC is synthesized via the typical eukaryotic CDP-choline pathway (Kent et al. 2004). Choline is converted to phosphocholine and then to CDP-choline by a LicCA fusion protein. Subsequently, PC is formed by the conversion of CDP-choline and DAG by the cholinephosphotransferase CPT. The most recently discovered bacterial PC synthesis pathway acts via a yeast-like acylation mechanism and has been described for the plant pathogen *X. campestris* and the human pathogens *Streptococcus pneumoniae*, *Streptococcus mitis*, and *Streptococcus oralis* (Moser et al. 2014; Joyce et al. 2019). Here, glycerophosphocholine (GPC) is esterified with two fatty acids from acyl-CoA to lyso-PC (LPC) and subsequently to PC (Moser et al. 2014).

In this study, we demonstrate different strategies, by which *E. coli* can be manipulated to produce PC and other methylated PE derivatives. Several approaches rely on the ectopic expression of genes from natural PC-producing organisms. In addition, we show that *E. coli* is able to produce PC by its own enzyme repertoire when LPC is provided.

## Material and methods

### Chemicals

2-(methylamino)ethanol (MMEA), 2-(ethylamino)ethanol (DMEA), 1-oleoyl-2-hydroxy-*sn*-glycero-3-phosphocholine sodium salt (LPC), 1,2-dioleoyl-*sn*-glycero-3-phosphoethanolamine (PE), 1,2-dioleoyl-*sn*-glycero-3-phospho-(1′-rac-glycerol) (PG), 1,2-dioleoyl-*sn*-glycero-3-phosphocholine (PC), 1′,3′-bis(1,2-dioleoyl-*sn*-glycero-3-phospho)-glycerol (CL), 1,2-dioleoyl-*sn*-glycero-3-phosphoethanolamine-*N*-methyl (MMPE), and 1,2-dioleoyl-*sn*-glycero-3-phosphoethanolamine-*N,N*-dimethyl (DMPE) were purchased from Avanti polar lipids. TLC silica gel 60 plates and molybdenum blue spray reagent were purchased from Sigma-Aldrich. All other chemicals used were of analytical grade and commercially available.

### Bacterial strains, plasmids, and oligonucleotides

Strains, plasmids, and oligonucleotides used in this study are listed in Tables S1 and S2. *Escherichia coli* was grown in Luria–Bertani (LB) (Bertani 1951) or M9 minimal (Miller 1972) medium at 37 °C. When required, kanamycin (50 µg/ml) or chloramphenicol (30 µg/ml) was added to the medium. *Escherichia coli* JM83 served as a cloning host. *Escherichia coli* BL21 (DE3) was used as a host for gene expression using pET-based or pCA24N expression plasmids. To construct pET24b vectors for recombinant overproduction of C-terminally His-tagged proteins, the corresponding genes were amplified via polymerase chain reaction (PCR) from chromosomal DNA, using appropriate primers (Table S2). The PCR products were integrated into pET24b using the indicated restriction sites (Table S2) resulting in the plasmids pBO807 (*B. diazoefficiens pmtA*), pBO2617 (*M. extorquens pmtA*), pBO2618 (*M. extorquens pmt2*), and pBO2628 (*P. fluorescens pcs*).

### Heterologous expression of pcs, pmt, or acyltransferase genes in E. coli

For the overexpression of genes of interest in *E. coli*, the corresponding plasmids were transferred into *E. coli* BL21 (DE3). Cells were cultivated at 37 °C for 18 h and from this, main cultures were inoculated at an initial optical density at a wavelength of 580 nm (OD_580_) of 0.1. Gene expression was induced with 0.1 mM isopropyl-*β*-D-thiogalactopyranoside (IPTG) at an OD_580_ of 0.5–0.8 and cells were subsequently incubated at 30 °C for 18 h. Finally, cultures were adjusted to an OD_580_ of 3 and 1 ml was harvested by centrifugation for lipid and protein analysis.

### Feeding E. coli with exogenous substrates

Cultivation of *E. coli* in the presence of ethanolamine derivatives or LPC was performed in M9 or LB medium, respectively. At the time of induction of gene expression, the cultures were supplemented with either 1 mM of monomethylethanolamine (MMEA), dimethylethanolamine (DMEA), or choline or with 0.5 mM of LPC solubilized in Triton X-100 (0.05%). Cultures were further incubated at 30 °C for 18 h and subsequently harvested by centrifugation. Samples for lipid and protein analysis were collected and analyzed as described below.

### PC formation in E. coli Δlplt crude extracts

*Escherichia coli* Δ*lplt* cells were harvested, washed, and adjusted to an OD_580_ of 60. The cell pellet was resuspended in 1 ml of lysis buffer (20 mM NaH_2_PO_4_ * 2H_2_O, 500 mM NaCl, 10% (v/v) glycerol, pH 8.0) and lysozyme (5 mg/ml) was added. Cells were incubated for 30 min on ice before disruption using a VialTweeter (Hielscher, Teltow, Germany) (6 sonication cycles, amplitude of 90, and duty cycle of 50). A total of 100 µl of crude extracts was then supplemented with 2 mM LPC solubilized in Triton X-100 (0.05%). Reactions were performed at 30 °C for 18 h. Total lipids were extracted and analyzed as described below.

### Lipid analysis

Lipids from resuspended cell pellets (in 100 µl distilled water) or from activity assays with crude extracts (100 µl) were isolated according to Bligh and Dyer (1959). The lower organic phase containing the lipids was evaporated in a speed vacuum centrifuge. Dried lipids were resuspended in methanol:chloroform (1:1) and analyzed by thin layer chromatography (TLC) using silica gel 60 plates (Merck). Total lipids were separated using *n*-propanol:propionic acid:chloroform:water (3:2:2:1) as mobile phase and phospholipids were visualized using molybdenum blue spray reagent (Dittmer and Lester 1964).

### Protein analysis

For protein analysis, cell pellets were resuspended in appropriate amounts of Laemmli sample buffer (Laemmli 1970). Protein samples were heated to 95 °C for 10 min, before equal amounts were separated by 12.5% sodium dodecyl sulfate polyacrylamide gel electrophoresis (SDS-PAGE). Proteins were stained with Coomassie blue.

## Results

### Biosynthesis of methylated PE derivatives by Pmts

**Fig. 3:**
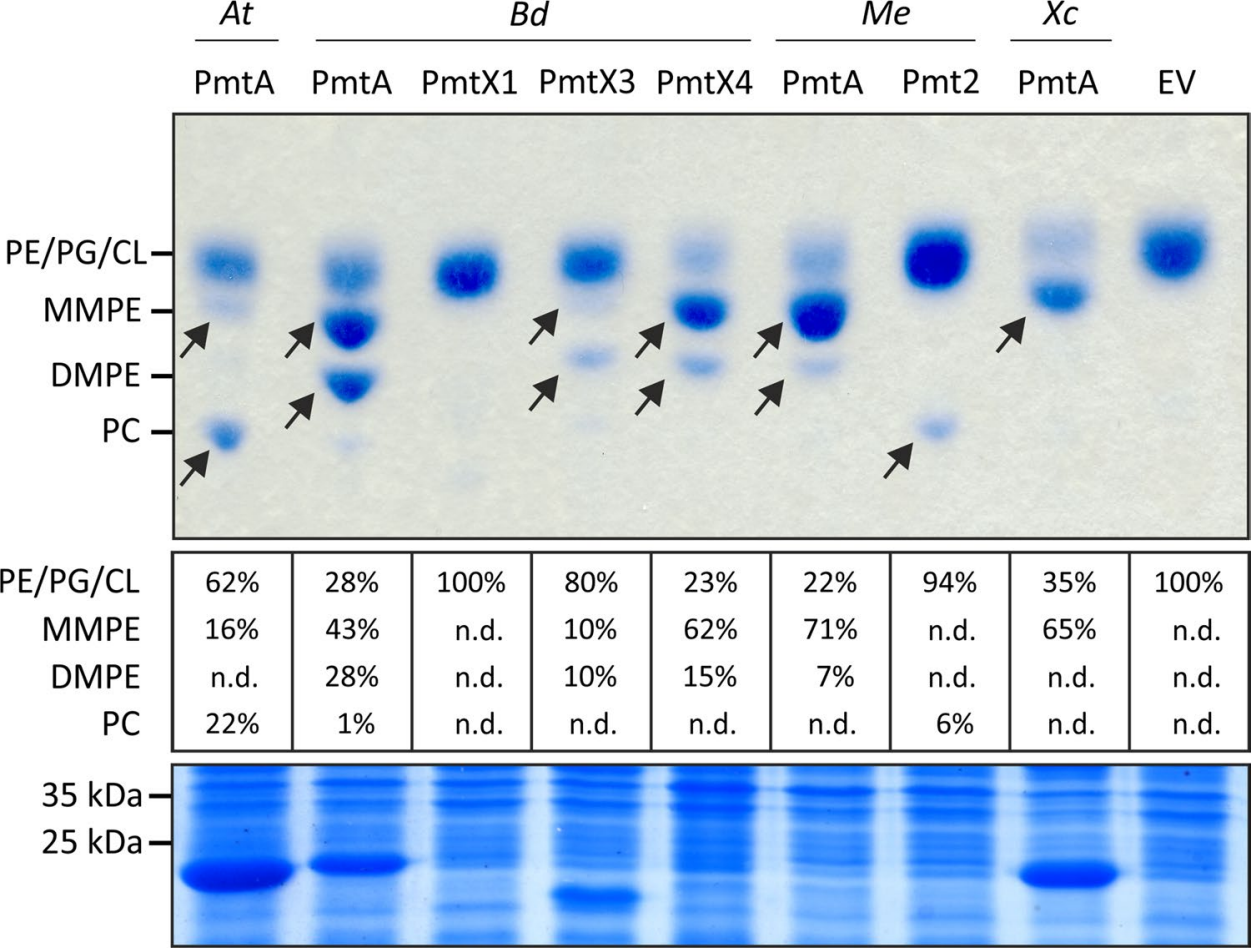
Biosynthesis of methylated PE derivatives upon heterologous expression of various bacterial *pmts* in *E. coli*. Expression of the respective genes in *E. coli* BL21 was induced using 0.1 mM IPTG and cells were subsequently incubated at 30 °C for 16 h. Cells were harvested and lipids were extracted and separated via thin layer chromatography. Phospholipids were then visualized using molybdenum blue spray reagent and the relative amount of MMPE, DMPE, and PC [%] was quantified by pixel counting, using the AlphaEase FC software. The position of commercially available C18:1 phospholipids is indicated. Arrows indicate formed products. Proteins were separated using SDS-PAGE. *At*: *A. tumefaciens*; *Bd*: *B. diazoefficiens*; *Me*: *M. extorquens*; *Xc*: *X. campestris*; EV: empty vector control; PE: phosphatidylethanolamine; PG: phosphatidylglycerol; CL: cardiolipin; MMPE: monomethyl-PE; DMPE: dimethyl-PE; PC: phosphatidylcholine; n.d.: not detected

Bacteria have various ways to produce PC. Thanks to absence of all the enzymes depicted in Fig. 2, *E. coli* has become a very popular host for their heterologous production and functional characterization (Klüsener et al. 2009; Hacker et al. 2008b; Moser et al. 2014; Kleetz et al. 2021). First, we compared the diverse substrate and product spectra of several bacterial Pmts when produced in *E. coli* (Fig. 3). The membrane of *E. coli* carrying the empty vector (EV) shows the typical profile of PE, PG, and CL. As shown previously (Wessel et al. 2006), the expression of *pmtA* from *A. tumefaciens* resulted in the accumulation of MMPE and PC (16% and 22% of the total phospholipids, respectively), whereas di-methylated DMPE was hardly detected (< 1%). The successful overexpression of *A. tumefaciens pmtA* was demonstrated by a prominent protein band at the calculated molecular size of ~ 23 kDa in the SDS gel (Fig. 3, lower panel). The situation in *B. diazoefficiens* is more complex (Figs. 2 and 3). Expression of *B. diazoefficiens pmtA* resulted in the synthesis of large amounts of MMPE and DMPE (43% and 28% of the total phospholipids, respectively), but only traces of PC (1% of the total phospholipids). *Bradyrhizobium diazoefficiens* PmtX1 preferably uses MMPE (which is absent in *E. coli*) as a substrate to produce DMPE and PC (Hacker et al. 2008b). Therefore, *pmtX1* expression in *E. coli* did not visibly alter the membrane composition (Fig. 3). It was shown previously that synthesis of large amounts of PC by PmtX1 in *E. coli* can be achieved by the co-expression of *pmtX1* and *pmtA*, which produces the necessary substrates MMPE and DMPE for PmtX1 (Hacker et al. 2008b). Heterologous expression of the two additional *B. diazoefficiens pmts* (*pmtX3* and *pmtX4*) led to accumulation of MMPE (10% and 65% of the total phospholipids, respectively) and DMPE (10% and 15% of the total phospholipids, respectively). As judged by SDS-PAGE, the four *B. diazoefficiens* Pmts were produced in different quantities, with large amounts of PmtA (~ 23 kDa) and PmtX3 (~ 21 kDa) and no visibly detectable protein for PmtX1 (~ 24 kDa) and PmtX4 (~ 25 kDa). The two Pmt candidates *pmtA* and *pmt2* of the *Methylobacterium extorquens*, an alpha-proteobacterium with an exceptionally adaptive lipidome living in the plant phyllosphere (Chwastek et al. 2020), have never been studied. Although both gene products (expected size ~ 24 kDa) were undetectable in *E. coli* crude extracts, they were able to convert PE to various methylated products (Fig. 3). *Methylobacterium extorquens* PmtA predominantly produced MMPE (71% of the phospholipids) and traces of DMPE (7% of the phospholipids) at the expense of PE, while Pmt2 produced PC (6% of its total phospholipids) without accumulation of the intermediates. The panel of Pmt enzymes from plant-associated bacteria was completed by PmtA from the pathogen *X. campestris*, which methylated *E. coli* PE to large amounts of MMPE (65% of the total phospholipids).

The results show that—with the help of recombinant enzymes—*E. coli* readily converts large amounts of its resident PE to methylated products. Interestingly, in several cases, the resulting re-arrangements in the phospholipid composition are substantial without having an obvious impact on *E. coli* growth and fitness (data not shown). As noted previously (Kleetz et al. 2021), the exact methylation products are impossible to predict based on sequence alignments of Pmt enzymes. They must be tested experimentally for each new candidate.

### Efficient biosynthesis of MMPE, DMPE, and PC by Pcs

**Fig. 4:**
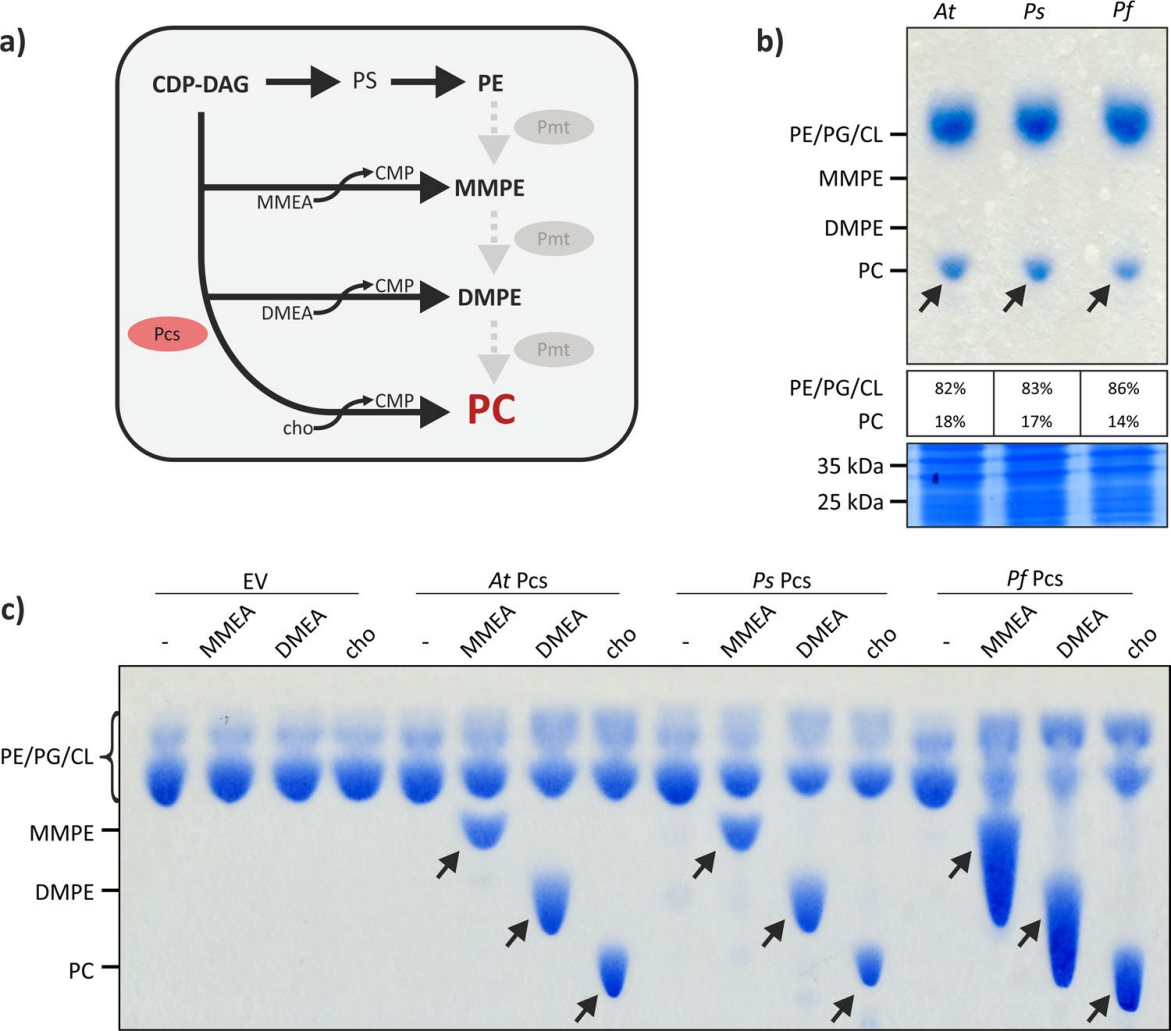
Biosynthesis of methylated PE derivatives in *E. coli* by PC synthases. (**a**) PC synthase (Pcs) pathway and alternative substrates utilized by Pcs. In addition to the naturally occurring PC synthesis from CDP-DAG and choline, Pcs also produces DMPE and MMPE using dimethylethanolamine (DMEA) and monomethylethanolamine (MMEA). MMPE and DMPE are common intermediates of the methylation pathway, as depicted in light gray. (**b**) and (**c**) *Escherichia coli* BL21 cells carrying the respective expression vectors were grown in LB medium (**b**) or M9 minimal medium (**c**) and gene expression induced using 0.1 mM IPTG. After incubation at 30 °C for 16 h, cells were harvested. Proteins were separated via SDS-PAGE and lipids were extracted and separated via TLC. Phospholipids were visualized using molybdenum blue spray reagent and the relative amount of PC [%] upon expression of the different *pcs* was quantified by pixel counting using the AlphaEase FC software. The position of commercially available C18:1 phospholipids is indicated. Arrows indicate formed products. (**c**) Synthesis of MMPE, DMPE, and PC in presence of MMEA, DMEA, or choline (cho). *Escherichia coli* was grown in M9 minimal medium and 1 mM of the respective substrate was added simultaneous to induction by IPTG. *At*: *A. tumefaciens*; *Ps*: *P. syringae*; *Pf*: *P. fluorescens*; PE: phosphatidylethanolamine; PG: phosphatidylglycerol; CL: cardiolipin; MMPE: monomethyl-PE; DMPE: dimethyl-PE; PC: phosphatidylcholine; CDP-DAG: cytidine diphosphate diacylglycerol; PS: phosphatidylserine; Pmt: phospholipid *N*-methyltransferase; CMP: cytidine monophosphate; EV: empty vector control; n.d.: not detected

Another common bacterial PC biosynthesis pathway is the condensation of CDP-DAG and choline catalyzed by Pcs (Fig. 4a). When grown in medium containing a choline source, such as LB medium, *E. coli* strains carrying the EV did not produce PC, whereas strains expressing *A. tumefaciens pcs*, *Pseudomonas syringae pcs*, or *Pseudomonas fluorescens pcs* produced PC in comparable amounts (14–18% of their total phospholipids) (Fig. 4b). At the same time, overproduction of these multitransmembrane proteins (~ 27 kDa) was not observed by SDS-PAGE analysis of *E. coli* cell extracts (Fig. 4b, lower panel). When *E. coli* cultures were grown in M9 minimal medium missing a choline source, only the regular phospholipid PE, PG, and CL were detected regardless of whether the strains contained an EV or *pcs* expression plasmids (Fig. 4c). When choline was supplemented to the medium, PC was synthesized by all three Pcs. Besides choline, Pcs enzymes have been described to accept monomethylethanolamine (MMEA) or dimethylethanolamine (DMEA) to produce MMPE and DMPE (Fig. 4a) (Aktas et al. 2014; Vasilopoulos et al. 2021). Here, we show that *E. coli* strains expressing *pcs* genes can be used to produce copious amounts of the valuable lipids MMPE, DMPE, and PC when the growth medium is supplemented with the low-cost compounds MMEA, DMEA, and choline, respectively (Fig. 4c).

### Synthesis of PC from LPC by acyltransferases

**Fig. 5:**
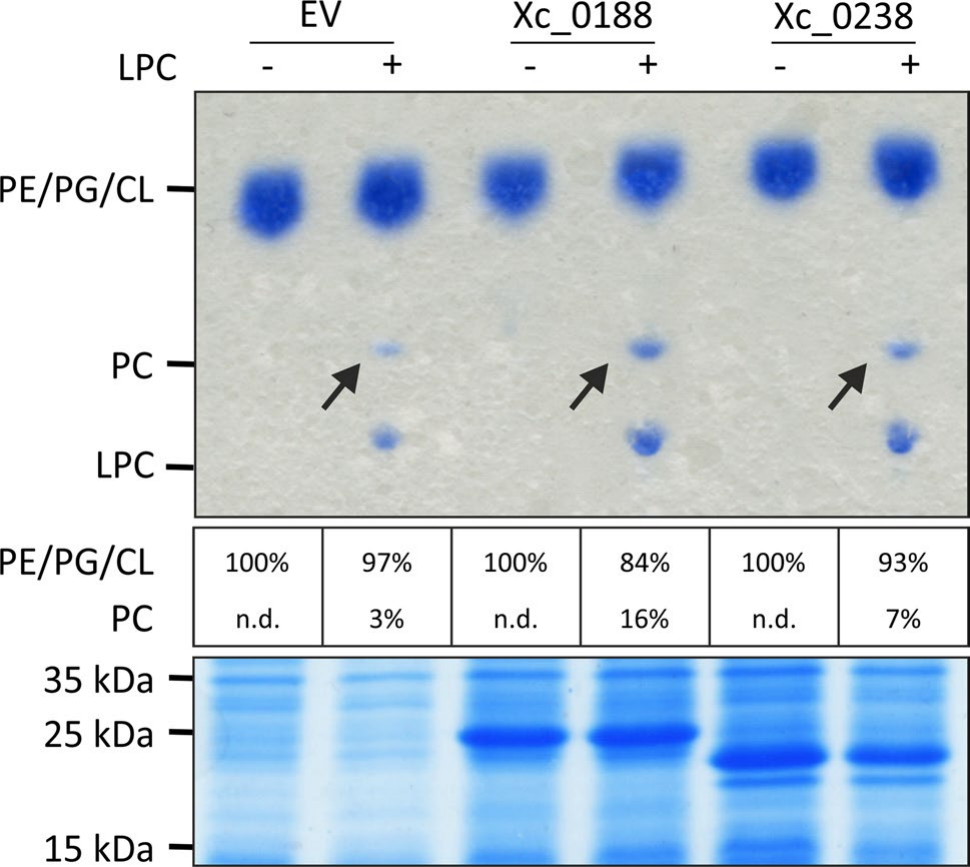
Acylation of LPC to PC by heterologous production of acyltransferases in *E. coli*. Expression of the respective genes *xc_0188* and *xc_0238* was induced using 0.1 mM IPTG and cells were subsequently incubated at 30 °C for 16 h. For PC synthesis, 0.1 mM LPC as Triton-mixed micelles was simultaneously supplemented. Proteins were separated via SDS-PAGE. Lipids were extracted from cell pellets and separated via TLC. Phospholipids were visualized using molybdenum blue spray reagent and the relative amount of PC [%] upon expression of the different acyltransferases was quantified by pixel counting using the AlphaEase FC software. The position of commercially available C18:1 phospholipids is indicated. Arrows indicate formed products. PE: phosphatidylethanolamine; PG: phosphatidylglycerol; CL: cardiolipin; PC: phosphatidylcholine; LPC: lyso-PC; EV: empty vector control; n.d.: not detected

An alternative pathway for PC biosynthesis has only recently been described in divergent bacteria such as the Gram-negative *X. campestris* and several Gram-positive *Streptococcus* species (Moser et al. 2014; Joyce et al. 2019). It involves the acylation of mono-acylated LPC to PC by acyltransferases. In *X. campestris*, this reaction is carried out by either of the two acyltransferases Xc_0188 and Xc_0238. Both proteins could be recombinantly produced in large amounts in *E. coli* and, in the absence of LPC, did not alter the bacterial membrane composition (Fig. 5, upper panel). When LPC was added, both *E. coli* strains producing either of the two acyltransferases synthesized PC (16% and 7% of the total phospholipids upon expression of *xc_0188* and *xc_0238*, respectively). Notably and in agreement with a previous report (Moser et al. 2014), *E. coli* cells carrying the EV also produced marginal amounts of PC from LPC (3% of the total phospholipids), which led us to search for the underlying endogenous PC-forming activity.

### PC biosynthesis by E. coli native proteins

**Fig. 6:**
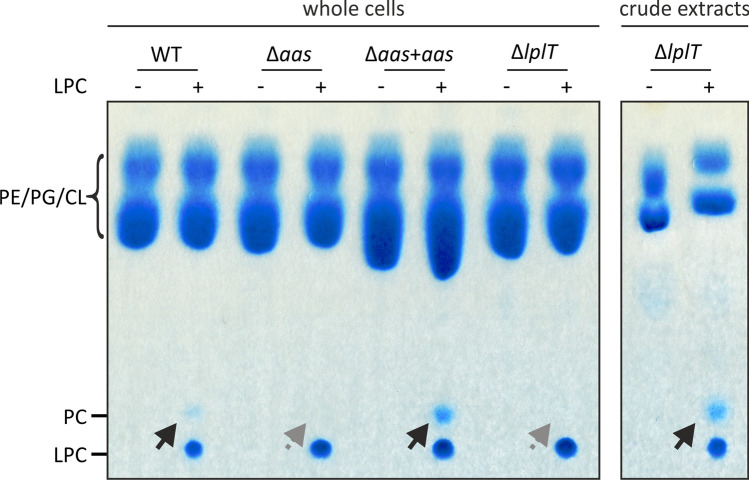
Native *E. coli* proteins responsible for PC biosynthesis from LPC. Screening of strains from the Keio knockout collection (Baba et al. 2006) revealed Aas and LplT to have an influence on PC biosynthesis in *E. coli*. The Keio wild type (WT) as well as the Δ*aas* and Δ*lplT* mutant were grown in the absence (−) and presence (+) of 0.1 mM LPC as Triton-mixed micelles. The Δ*aas* mutant was additionally complemented by an *aas* expression plasmid (Δ*aas* + *aas*). Here, gene expression was induced using 0.1 mM IPTG. Cells were subsequently incubated in presence or absence of LPC at 30 °C for 16 h. PC synthesis of the Δ*lplT* mutant was additionally tested in crude extracts in the presence of 2 mM LPC as Triton-mixed micelles. From all conditions, cells were subsequently harvested and lipids were extracted, separated via thin layer chromatography and phospholipids visualized using molybdenum blue spray reagent. The position of commercially available C18:1 phospholipids is indicated. Black arrows indicate formed products, while gray, dashed arrows indicate the absence of products. PE: phosphatidylethanolamine; PG: phosphatidylglycerol; CL: cardiolipin; PC: phosphatidylcholine; LPC: lyso-PC

For the identification of the *E. coli* specific proteins involved in the acylation of LPC (Fig. 5), strains from the Keio knockout collection (Baba et al. 2006) were screened for their ability to produce PC in the presence of LPC. Candidates were chosen based on annotated acyltransferase or acetyltransferase activities of the proteins or their function in lysophospholipid metabolism (Table S3). The corresponding *E. coli* knockout mutants were grown in the absence or presence of LPC and the capacity to produce PC was analyzed by TLC. Under all tested conditions, PC formation in *E. coli* was strictly dependent on the presence of LPC. Out of the 81 candidates, only deletion of the genes *aas* (coding for the acyltransferase-acyl carrier protein synthetase Aas) or *lplt* (coding for the lysophospholipid transporter Lplt) abolished PC biosynthesis (Fig. 6). The phenotype of the Δ*aas* strain was readily restored upon complementation with an *aas* expression plasmid. The Δ*lplT* mutant was unable to produce PC in intact *E. coli* cells. However, when the cells were disrupted and LPC was added to crude extract, PC formation was restored suggesting that LplT is involved in the transport of LPC across the membrane(s).

## Discussion

The membrane composition of *E. coli* is often believed to be exemplary for bacteria. Indeed, many of the principles and pathways of membrane biogenesis identified in pioneering work apply to numerous related and non-related bacterial species. However, advances in analytical instrumentation in recent years have uncovered a plethora of lipid species in the bacterial membrane, many of them with yet unknown functions. It has become evident that the membrane composition is highly flexible and strongly depends on environmental conditions and substrate availabilities. Here, we report how *E. coli* membranes can be manipulated to contain significant amounts of non-native lipids.

**Fig. 7:**
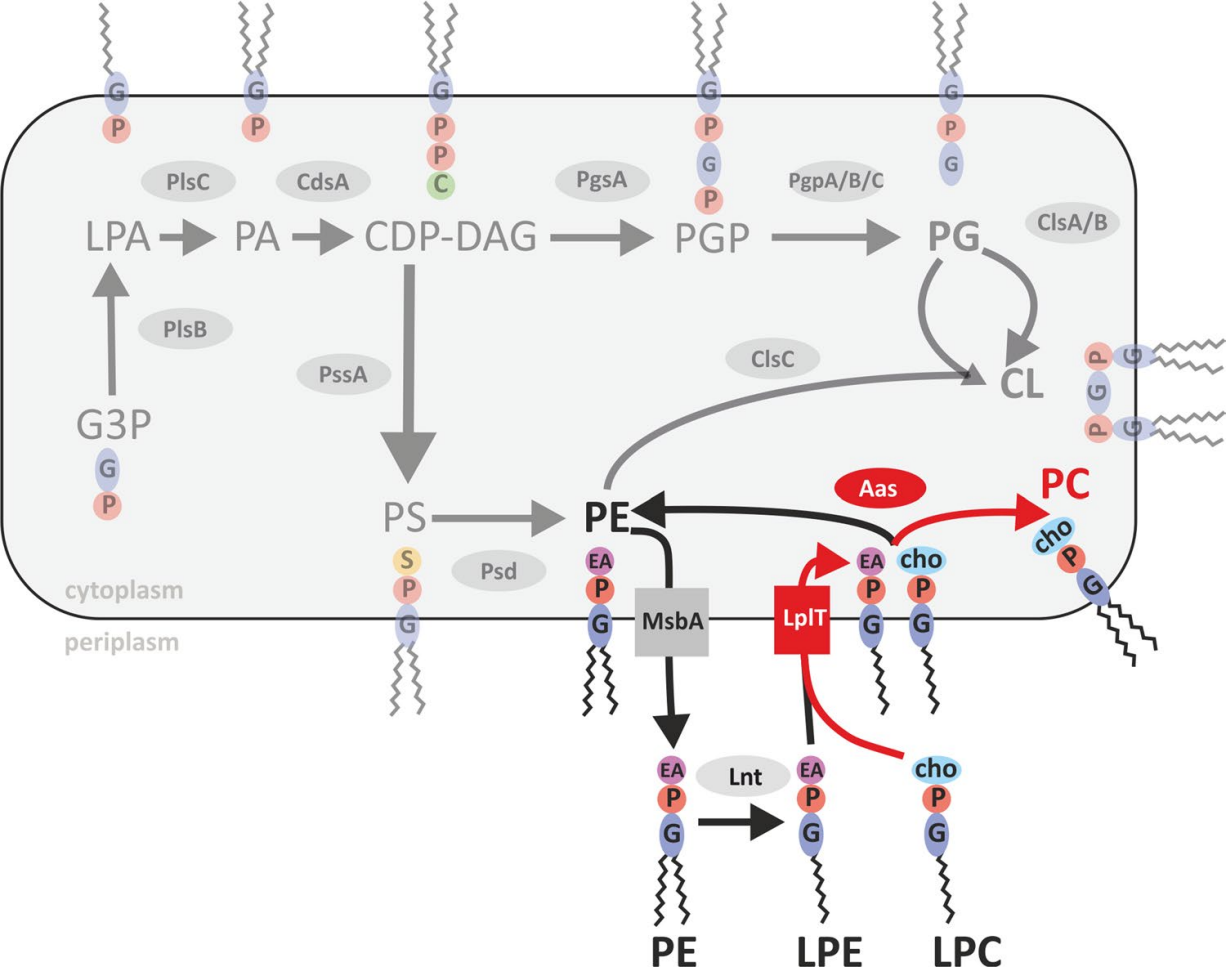
Extended overview of phospholipid-synthesis pathways in *E. coli* including PC biosynthesis. Details on the individual enzyme reactions and phospholipid structures are given in the text and Fig. 1. The LplT/Aas system responsible for the transport and acylation of LPC is indicated in red. LptT: lysophospholipid transporter; Aas: acyltransferase-acyl carrier protein synthetase; MslbA: lipopolysaccharide transporter; Lnt: apolipoprotein *N*-acyltransferase

It previously went largely unnoticed that *E. coli* has the intrinsic potential to produce PC. The lysophospholipid transporter LplT in concert with the acyltransferase-acyl carrier protein synthetase Aas are able to promote PC biosynthesis in the presence of LPC (Fig. 6). The LplT/Aas system is common among Gram-negative bacteria (Harvat et al. 2005; Lin et al. 2018a). Some of them such as *B. diazoefficiens* and *Mesorhizobium loti* produce a single protein, with Aas and Lplt activities fused in the same polypeptide chain. In *E. coli*, the *lplT* gene is encoded in the same operon as the *aas* gene; however, LplT and Aas are synthesized as two distinct proteins (Harvat et al. 2005). This LplT/Aas system is responsible for flipping lysophospholipids across the inner membrane from the periplasm to the cytoplasmic site. Lipids or the lipopolysaccharide component lipid A can be transported to the periplasm by the lipopolysaccharide transporter MsbA, where the apolipoprotein *N*-acyltransferase Lnt deacylates these molecules and produces lysolipids (Fig. 7) (Zhou et al. 1998; Hillmann et al. 2011). Aas catalyzes the acylation of lysophospholipids to the fully acylated glycerophospholipids using acyl-ACP as fatty acid donor. The LplT/Aas system mainly uses LPE and LPG as substrates emerging from outer membrane lipoprotein maturation (Lin et al. 2016). Whether LPC is an accepted substrate is a disputed question in the literature. Lin et al. (2016) examined potential substrates of *Klebsiella pneumoniae* LplT in vitro as well as in *E. coli* spheroplasts expressing *K. pneumoniae lplT*. These studies confirmed LPE and LPG as preferred substrates but did not observe activity using LPC. These results were supported by computational docking of *K. pneumoniae* LplT to different substrates, again showing LPC to be unfavorable (Lin et al. 2018b). Other studies reported LPC uptake and acylation mediated by the *E. coli* LplT/Aas system (Harvat et al. 2005; Hsu et al. 1991; Jackowski et al. 1994). However, the efficiency of LPC acylation compared to LPE acylation by Aas was 40 times lower (Hsu et al. 1991). LPC acylation reported by Hsu et al. (1991) as well as in our study were observed in *E. coli* in vivo experiments using relatively high concentrations of the substrate (0.1 mM and 1 mM respectively). Although the concentration of LPC in body fluids can be similarly high at around 0.15 mM (Rabini et al. 1994), the amount of free LPC is assumed to be minute (Riederer et al. 2010). Together with PC, LPC makes up for 60–90% of all lipids in the intestinal mucus (Bernhard et al. 1995; Ehehalt et al. 2004), which is the typical habitat of *E. coli*. It is thus feasible that *E. coli* takes up and acylates low amounts of LPC in its physiological environment. Based on our and the previously reported results, we propose to add LPC to the accepted substrates of the LplT/Aas system in *E. coli* (Fig. 7). At present, it remains unclear whether PC is of physiological relevance for *E. coli*. PC in bacteria has been demonstrated to affect various processes including motility or stress resistance (Medeot et al. 2010; Wessel et al. 2006; Klüsener et al. 2009). More importantly, PC has been shown to be of great importance for the pathogenic or symbiotic interaction of many bacteria with their eukaryotic hosts (e.g., Aktas et al. (2010)). Transcriptomic studies in *A. tumefaciens* and *B. diazoefficiens* further revealed a strong impact of PC deficiency on the expression of many genes encoding membrane-associated and transport-associated proteins (Klüsener et al. 2010; Hacker et al. 2008a). Hence, while the specific functional role of PC in *E. coli* is unexplored thus far, it is feasible that PC biosynthesis in an LPC-rich environment triggers a response in *E. coli.*

Although large amounts of PC in the membrane of standard *E. coli* expression strains will not be reached, the possibility to externally supply LPC to growing cells broadens the options to tailor their membrane composition without any genetic manipulation. In case that higher PC (or MMPE or DMPE) concentrations in the *E. coli* membrane are desired, the heterologous expression of a variety of enzymes offers a range of options (Table 1). Similar to PC biosynthesis by native *E. coli* proteins, two acyltransferases from *X. campestris* are able to acylate LPC to PC upon heterologous expression of the respective genes (Fig. 5) (Moser et al. 2014). This yeast-like acylation pathway also yields low amounts of PC, which amounts to ~ 6% in the *X. campestris* membrane (Moser et al. 2014). The plant pathogen further codes for an interesting PmtA enzyme, which specifically produces MMPE, but not DMPE and PC (Fig. 3) (Moser et al. 2014). Since the lipids produced by Pmts vary strongly between the enzymes from different bacteria, one can choose from different options (Fig. 3, Table 1). Producing PC in *E. coli* with the help of Pmts is attractive because the methylation pathway does not depend on any external substrate, since PE as well as the methyldonor SAM are naturally available in *E. coli*. Thus, *pmt-*expressing cells can simply be grown in complex media such as LB. This also applies to PC production by recombinant Pcs enzymes as LB medium is rich in choline (Fig. 4). Choline needs to be supplemented only when minimal media like M9 are used. It has previously been reported that *A. tumefaciens* and *P. syringae* Pcs are able to convert MMEA and DMEA to MMPE and DMPE, respectively (Aktas et al. 2014; Vasilopoulos et al. 2021), and our results show that this might be a conserved feature of Pcs enzymes. The supplementation of low-cost MMEA, DMEA, or choline allowed for the accumulation of large amounts of the valuable lipids MMPE, DMPE, or PC in the membrane without eliciting any obvious growth abnormalities. This highlights the remarkable plasticity of the *E. coli* membrane, which has many possible applications in a biotechnological and biomedical context (Pichler and Emmerstorfer-Augustin 2018).

**Table 1:** Overview of enzymes from diverse bacteria that form PC in *E. coli*. *PE* phosphatidylethanolamine; *MMPE* monomethyl-PE; *DMPE* dimethyl-PE; *PC* phosphatidylcholine; *MMEA* monomethylethanolamine; *DMEA* dimethylethanolamine; *SAM S*-adenosyl methionine; *CDP-DAG* cytidine diphosphate diacylglycerol; *ACP* acyl carrier protein; *CoA* coenzyme A

	**Name**	**Host organism**	**Lipid products in *E. coli***	**Required substrates**		**Reference**
**Phospholipid *N*-methyltransferases**	PmtA	*A. tumefaciens*	MMPE DMPE PC	PE	SAM	Klüsener et al. (2009)
	PmtA	*B. diazoefficiens*	MMPE	PE		Hacker et al. (2008b) Minder et al. (2001)
	PmtX1		DMPE PC	MMPE		
	PmtX3		MMPE DMPE	PE		
	PmtX4		MMPE DMPE	PE		
	PmtA	*M. extorquens*	MMPE DMPE	PE		This study Moser et al. (2014)
	Pmt2		PC	MMPE DMPE		
	PmtA	*X. campestris*	MMPE	PE		Moser et al. (2014)
**PC synthases**	Pcs	*A. tumefaciens*	MMPE DMPE PC	MMEA DMEA Choline	CDP-DAG	Klüsener et al. (2009) Aktas et al. (2014)
	Pcs	*P. syringae*				This study Vasilopoulos et al. (2021)
	Pcs	*P. fluorescens*				This study
**Acyltransferases**	Xc_0188	*X. campestris*	PC	LPC	Acyl-ACP/Acyl-CoA	Moser et al. (2014)
	Xc_0238					
	Aas	*E. coli*				This study Hsu et al. (1991) Harvat et al. (2005)

Although *E. coli* is a versatile and widely applied heterologous host in research and biotechnology (Lee and Lee 2003; Idalia and Bernardo 2017; Cronan 2014; Rosano et al. 2019), it has its limitations for instance when it comes to membrane proteins (Opekarová and Tanner 2003). Modification of the fatty acid composition of the expression host *E. coli* CP41(DE3) is one promising approach for the improved heterologous production of membrane proteins (Kanonenberg et al. 2019). For eukaryotic membrane proteins, the *E. coli* membrane may also be unfavorable in terms of headgroup composition because of the absence of the most abundant eukaryotic phospholipid PC. For instance, the bovine PC-transfer protein was produced in an inactive form in *E. coli* (Brouwer et al. 2001). Renaturation from inclusion bodies using a buffer containing PC led to correct folding and activity. Interestingly, recombinant production of the bovine PC-transfer protein in PC-synthesizing *E. coli* cells resulted in active protein. In another example, the activity of the bacterial multidrug transporter HorA was positively influenced when reconstituted in membranes containing PC instead of PE, as the orientation of a transmembrane helix was affected (Gustot et al. 2010). The transphosphatidylation reaction of *E. coli* ClsA has also been shown to be strictly dependent on the presence of foreign PC (Jeucken et al. 2018). These and many other examples demonstrate how important it might be for various applications to manipulate the *E. coli* membrane to contain PC or other methylated phospholipids.

The integration of MMPE, DMPE, or PC into *E. coli* membranes might have other possible advantages in biotechnological applications. Methylated PE derivatives, especially PC, were induced in response to lower temperatures in *M. extorquens* (Chwastek et al. 2020). Together with a higher degree of saturation, the larger headgroup of methylated PE derivatives increased membrane fluidity, thus adapting the cells to lower temperatures (Chwastek et al. 2020; Klose et al. 2013). It is therefore conceivable that PC-producing *E. coli* cells are better suited for biotechnological processes performed at lower temperatures, for example with cold-adapted proteases from psychrophilic bacteria that have numerous applications in food and detergent industry (Furhan 2020). In another application, *E. coli* expressing a *pcs* gene has successfully been used for the production of selectively deuterated PC (Maric et al. 2015). Prior to this study, deuterated phospholipids were chemically synthesized, sometimes requiring complex organic synthesis (Bragina and Chupin 1997). Deuterated phospholipids have numerous applications in mass spectrometry, NMR, or the study of membrane proteins (Hagn et al. 2013; Akesson et al. 2012; Rohwedder 1985). Feeding of MMEA or DMEA to *pcs-*expressing *E. coli* cells or the expression of *pmt* genes offers the possibility to produce not only deuterated PC, but also MMPE and DMPE species.

The microbial production of high-value lipids has been recognized as one of the great opportunities in white biotechnology in the twenty-first century (Garay et al. 2014). The ease by which *E. coli* can be manipulated either by substrate feeding or by genetic engineering to produce a range of otherwise low-abundant or non-natural phospholipids offers many not yet fully explored possibilities in the future.

## Supplementary Information

Below is the link to the electronic supplementary material.

Supplementary file1 (PDF 354 KB)

## Data Availability

The datasets generated during and/or analyzed during the current study are available from the corresponding author on reasonable request.
