# Supplementary material for: Recombinant and endogenous ways to produce methylated phospholipids in Escherichia coli
Source: Appl Microbiol Biotechnol. 2021 Oct 28;105(23):8837–51. doi: 10.1007/s00253-021-11654-8 (PMC8590670; doi:10.1007/s00253-021-11654-8)
Supplement: Supplementary file 1 — Supplementary file1 (PDF 354 KB) [file 253_2021_11654_MOESM1_ESM.pdf]

Applied Microbiology and Biotechnology

Supplementary material

Recombinant and endogenous ways to produce methylated phospholipids in *Escherichia coli*

Julia Kleetz*, Georgios Vasilopoulos*, Simon Czolkoss, Meriyem Aktas, Franz Narberhaus#

Microbial Biology, Faculty of Biology and Biotechnology, Ruhr University Bochum, Germany

*JK and GV contributed equally to this study

#For correspondence: e-mail franz.narberhaus@rub.de; Tel. (+49) (234) 32 23100; Fax (+49) (234) 32 14620

Table S1 Bacterial strains and plasmids used in this study

Strain or plasmid	Characteristics	Reference or source
**Strain**		
Escherichia coli JM83	Cloning host	Vieira and Messing (1982)
Escherichia coli BL21 (DE3)	Expression host	Studier and Moffatt (1986)
Escherichia coli K12 (BW25113)	Keio collection "wild-type"	Baba et al. (2006)
Escherichia coli K12 (BW25113) Δ*aas*	Derivative of BW25113, deletion of *aas* gene	Baba et al. (2006)
Escherichia coli K12 (BW25113) Δ*lplt*	Derivative of BW25113, deletion of *lplt* gene	Baba et al. (2006)
**Plasmid**		
pET24b	Km^R, vector for overproduction of His-tagged proteins	Novagen, Darmstadt, Germany
pET28b	Km^R, vector for overproduction of His-tagged proteins	Novagen, Darmstadt, Germany
pCA24N	Cm^R, vector for overproduction of His-tagged proteins	Kitagawa et al. (2005)
pBO0832	pET28b derivative carrying *A. tumefaciens pmtA*	Aktas and Narberhaus (2009)
pBO807	pET24b derivative carrying *B. diazoefficiens pmtA*	This study
pCCS20	pET9a derivative carrying *B. diazoefficiens pmtX1*	Hacker et al. (2008)
pCCS37	pET9a derivative carrying *B. diazoefficiens pmtX3*	Hacker et al. (2008)
pBO0234	pET24b derivative carrying *B. diazoefficiens pmtX4*	Hacker et al. (2008)
pBO2617	pET24b derivative carrying *M. extorquens pmtA*	This study
pBO2618	pET24b derivative carrying *M. extorquens pmt2*	This study
pBO2604	pET28b derivative carrying *X. campestris pmtA*	Moser et al. (2014)
pBO803	pET24b derivative carrying *A. tumefaciens pcs*	Aktas et al. (2014)
pBO2614	pET24b derivative carrying *P. syringae pcs*	Vasilopoulos et al. (2021)
pBO2628	pET24b derivative carrying *P. fluorescens pcs*	This study
pBO3222	pET28b derivative carrying *xc_0188*	Moser et al. (2014)
pBO3273	pET28b derivative carrying *xc_0238*	Moser et al. (2014)
pCA24N_*aas*	pCA24N derivative carrying *E. coli aas*	Kitagawa et al. (2005)

Table S2 Oligonucleotides used in this study

Oligonucleotide	Sequence (5 → 3)[a]
B. diazoefficiens pmtA	
pmtA_Bd_NdeI_fw	GGA ATT CCA TAT GCC ATT GCC ATC GTC CGC GC
pmtA_Bd_XhoI_rv	CCG CTC GAG ATC CTT GCG ATA CAC CCA GAC GC
M. extorquens pmtA	
pmtA_Me_NdeI_fw	GGA ATT CCA TAT GCC GCC GCT TCG CCG
pmtA_Me_SalI_rv	CCG GTC GAC TCA AAG CTT GAC CGT GCC GGC CT
M. extorquens pmt2	
pmt2_Me_NdeI_fw	GGA ATT CCA TAT GCT GAG CGA GCG GGC
pmt2_Me_SalI_rv	CCG GTC GAC TCA AAG CTT GCC GCG CTT GCG CA
P. fluorescens pcs	
Pcs_Pf_NdeI_fw	GGA ATT CCA TAT GAT ATC GAC CCT GCA
Pcs_Pf_HindIII_rv	CCC AAG CTT TCA CTC GAG GCT GGC CTT GCT GA

[a] *Enzyme restriction sites are underlined*

Table S3 List of strains from the *E. coli* Keio knockout collection (Baba et al. 2006) that were screened for LPC conversion to PC

Gene name	Gene number	described protein function	LPC to PC conversion by the deletion mutant?
argA	JW2786	Amino-acid acetyltransferase	yes
astA	JW1736	Arginine *N*-succinyltransferase	yes
cysE	JW3582	Serine acetyltransferase	yes
metA	JW3973	Homoserine *O*-succinyltransferase	yes
paaE	JW1387	1,2-phenylacetyl-CoA epoxidase, subunit E	yes
paaJ	JW1392	3-oxoadipyl-CoA/3-oxo-5,6-dehydrosuberyl-CoA thiolase	yes
speG	JW1576	Spermidine *N*-acetyltransferase	yes
wcaB	JW2043	Putative colanic acid biosynthesis acetyltransferase	yes
leuA	JW0073	2-isopropylmalate synthase, acetyl-CoA *C*-acetyltransferase activity	yes
pflB	JW0886	Formate acetyltransferase 1	yes
pflD	JW3923	Formate acetyltransferase 2	yes
ybiW	JW0807	Formate C-acetyltransferase/glycerol dehydratase family glycyl radical enzyme	yes
cheY	JW1871	Chemotaxis protein, protein acetylation activity	yes
maeB	JW2447	NADP-dependent malic enzyme, acyl group transferase activity	yes
pflA	JW0885	Formate-*C*-acetyltransferase-activating enzyme 1	yes
pflC	JW3924	Pyruvate formate-lyase 2-activating enzyme/Formate-*C*-acetyltransferase-activating enzyme 2	yes
rffC	JW5597	dTDP-fucosamine acetyltransferase	yes
yihG	JW3834	Probable acyltransferase	yes
yiiD	JW3859	Probable acyltransferase	yes
yjaB	JW3972	Peptidyl-lysine *N*-acetyltransferase	yes
yjdJ	JW4088	*N*-acetyltransferase domain-containing protein	yes
elaA	JW2262	*N*-acetyltransferase domain-containing protein	yes
sufI	JW2985	Cell division protein	yes
ygiH	JW3031	Probable glycerol-3-phosphate acyltransferase/Lysophosphatidic acid synthase	yes
yhbS	JW3125	Uncharacterized *N*-acetyltransferase	yes
yhhK	JW3424	PanD regulatory factor, acetyl-CoA binding, *N*-acetyltransferase activity	yes

yhhY	JW3405	L-amino acid *N*-acetyltransferase (L-methionine *N*-acetyltransferase)	yes
yiaC	JW3519	Peptidyl-lysine *N*-acetyltransferase	yes
yiaH	JW3533	*O*-acetyltransferase	yes
aas	JW2804	2-acylglycerophosphoethanolamine acyltransferase	no
atoB	JW2218	Acetyl-CoA acetyltransferase	yes
fabH	JW1077	3-oxoacyl-[acyl-carrier-protein] synthase 3	yes
maa	JW0448	Maltose *O*-acetyltransferase	yes
mdoC	JW1034	Glucans biosynthesis protein C, succinyltransferase activity	yes
ycfT	JW1101	Protein with transferase activity, transferring acyl groups other than amino-acyl groups	yes
ydcK	JW1424	Uncharacterized acetyltransferase	yes
fabF	JW1081	3-oxoacyl-[acyl-carrier-protein] synthase 2	yes
yafP	JW0224	Uncharacterized *N*-acetyltransferase	yes
yfiD	JW2563	Autonomous glycyl radical cofactor, formate C-acetyltransferase activity	yes
ydcS	JW1435	Bifunctional polyhydroxybutyrate synthase, acyl group transferase activity	yes
yfiQ	JW2568	Peptidyl-lysine *N*-acetyltransferase	yes
ypeA	JW2427	Acetyltransferase	yes
aat	JW0868	Leucyl/phenylalanyl-tRNA--protein transferase, amino-acyl group transferase activity	yes
rimJ	JW1053	[Ribosomal protein S5]-alanine *N*-acetyltransferase	yes
rimL	JW1423	Ribosomal-protein-serine acetyltransferase	yes
aceF	JW0111	Dihydrolipoyllysine-residue acetyltransferase	yes
sucB	JW0716	Dihydrolipoyllysine-residue succinyltransferase component of 2-oxoglutarate dehydrogenase complex	yes
pta	JW2294	Phosphate acetyltransferase	yes
aceE	JW0110	Pyruvate dehydrogenase E1 component, pyruvate dehydrogenase (acetyl-transferring) activity	yes
fadA	JW5578	3-ketoacyl-CoA thiolase/Acetyl-CoA acyltransferase	yes
nrdG	JW4196	Anaerobic ribonucleoside-triphosphate reductase-activating protein, [formate-*C*-acetyltransferase]-activating enzyme activity	yes
citC	JW0610	[Citrate [pro-3S]-lyase] ligase, *N*-acyltransferase activity	yes
ddg	JW2375	Lipid A biosynthesis palmitoleoyltransferase	yes
phnO	JW4054	Aminoalkylphosphonate *N*-acetyltransferase	yes

wbbJ	JW2018	Putative lipopolysaccharide biosynthesis *O*-acetyl transferase	yes
lacA	JW0333	Acetyl-CoA:galactoside 6-*O*-acetyltransferase	yes
tdcE	JW5522	Keto-acid formate acetyltransferase	yes
lpxM	JW1844	Lipid A biosynthesis myristoyltransferase	yes
parC	JW2987	DNA topoisomerase 4 subunit A	yes
kbl	JW3592	2-amino-3-ketobutyrate coenzyme A ligase (Glycine acetyltransferase)	yes
ybiY	JW0808	Putative [Formate-C-acetyltransferase]-activating enzyme	yes
ggt	JW3412	Glutathione hydrolase proenzyme, acyltransferase activity	yes
ypfI	JW2459	tRNA(Met) cytidine acetyltransferase	yes
yaiX	JW0350	Putative uncharacterized acetyltransferase	yes
yfcY	JW2339	3-ketoacyl-CoA thiolase/Acetyl-CoA acyltransferase	yes
yjhQ	JW4269	Uncharacterized *N*-acetyltransferase	yes
entE	JW0586	Enterobactin synthase component E, acyltransferase activity	yes
wcaF	JW2039	Putative colanic acid biosynthesis acetyltransferase	yes
rimI	JW4335	[Ribosomal protein S18]-alanine *N*-acetyltransferase	yes
nhoA	JW1458	Arylamine *N*-acetyltransferase	yes
eutI	JW2442	Ethanolamine utilization protein	yes
plsX	JW5156	Acyl-[acyl-carrier-protein]--phosphate acyltransferase	yes
yqeF	JW5453	Probable acetyl-CoA acetyltransferase	yes
yjgM	JW5758	Uncharacterized *N*-acetyltransferase	yes
yncA	JW5233	L-amino acid *N*-acyltransferase	yes
crcA	JW0617	Lipid A palmitoyltransferase PagP	yes
yghO	JW5848	Contains Acyl-CoA-acyltransferase domain	yes
lipB	JW5089	Octanoyl-[acyl-carrier-protein]-protein *N*-octanoyltransferase	yes
lpxL	JW1041	Lipid A biosynthesis lauroyltransferase	yes
lplT	JW2803	Lysophospholipid transporter	no

References

Aktas M, Köster S, Kizilirmak S, Casanova JC, Betz H, Fritz C, Moser R, Yildiz Ö, Narberhaus F (2014) Enzymatic properties and substrate specificity of a bacterial phosphatidylcholine synthase. FEBS J 281:3523–3541. 10.1111/febs.12877

Aktas M, Narberhaus F (2009) *In vitro* characterization of the enzyme properties of the phospholipid *N*-methyltransferase PmtA from *Agrobacterium tumefaciens*. J Bacteriol 191:2033–2041. 10.1128/JB.01591-08

Baba T, Ara T, Hasegawa M, Takai Y, Okumura Y, Baba M, Datsenko KA, Tomita M, Wanner BL, Mori H (2006) Construction of *Escherichia coli* K-12 in-frame, single-gene knockout mutants: the Keio collection. Mol Syst Biol 2:2006.0008. 10.1038/msb4100050

Hacker S, Sohlenkamp C, Aktas M, Geiger O, Narberhaus F (2008) Multiple phospholipid *N*-methyltransferases with distinct substrate specificities are encoded in *Bradyrhizobium japonicum*. J Bacteriol 190:571–580. 10.1128/JB.01423-07

Kitagawa M, Ara T, Arifuzzaman M, Ioka-Nakamichi T, Inamoto E, Toyonaga H, Mori H (2005) Complete set of ORF clones of *Escherichia coli* ASKA library (a complete set of E K-12 ORF archive): unique resources for biological research. DNA Res 12:291–299. 10.1093/dnares/dsi012

Moser R, Aktas M, Narberhaus F (2014) Phosphatidylcholine biosynthesis in *Xanthomonas campestris via* a yeast-like acylation pathway. Mol Microbiol 91:736–750. 10.1111/mmi.12492

Studier F, Moffatt BA (1986) Use of bacteriophage T7 RNA polymerase to direct selective high-level expression of cloned genes. J Mol Biol 189:113–130. 10.1016/0022-2836(86)90385-2

Vasilopoulos G, Moser R, Petersen J, Aktas M, Narberhaus F (2021) Promiscuous phospholipid biosynthesis enzymes in the plant pathogen *Pseudomonas syringae*. Biochim Biophys Acta Mol Cell Biol Lipids:158926. 10.1016/j.bbalip.2021.158926

Vieira J, Messing J (1982) The pUC plasmids, an M13mp7-derived system for insertion mutagenesis and sequencing with synthetic universal primers. Gene 19:259–268. 10.1016/0378-1119(82)90015-4
